# Identification and Comparison of Hyperglycemia-Induced Extracellular Vesicle Transcriptome in Different Mouse Stem Cells

**DOI:** 10.3390/cells9092098

**Published:** 2020-09-15

**Authors:** Grace Huang, Venkata Naga Srikanth Garikipati, Yan Zhou, Cynthia Benedict, Steven R. Houser, Walter J. Koch, Raj Kishore

**Affiliations:** 1 Center for Translational Medicine, Lewis Katz School of Medicine, Temple University, Philadelphia, PA 19140, USA; tug78490@temple.edu (G.H.); cindy.benedict@temple.edu (C.B.); walter.koch@temple.edu (W.J.K.); 2 Department of Emergency Medicine, Dorothy M Davis Heart and Lung Research Institute, Wexner Medical School, The Ohio State University, Columbus, OH 43210, USA; Venkata.garikipati@osumc.edu; 3 Biostatistics and Bioinformatics Facility, Fox-Chase Cancer Center, Temple Health, Philadelphia, PA 19140, USA; yan.zhou@fccc.edu; 4 Cardiovascular Research Center, Lewis Katz School of Medicine, Temple University, Philadelphia, PA 19140, USA; srhouser@temple.edu; 5 Department of Pharmacology, Lewis Katz School of Medicine, Temple University, Philadelphia, PA 19140, USA

**Keywords:** extracellular vesicles/exosomes, stem cells, hyperglycemia, RNA sequencing

## Abstract

Extracellular vesicles (EVs) derived from stem /progenitor cells harbor immense potential to promote cardiomyocyte survival and neovascularization, and to mitigate ischemic injury. However, EVs’ parental stem/progenitor cells showed modest benefits in clinical trials, suggesting autologous stem cell/EV quality might have been altered by stimuli associated with the co-morbidities such as hyperglycemia associated with diabetes. Hyperglycemia is a characteristic of diabetes and a major driving factor in cardiovascular disease. The functional role of stem/progenitor cell-derived EVs and the molecular signature of their secreted EV cargo under hyperglycemic conditions remain elusive. Therefore, we hypothesized that hyperglycemic stress causes transcriptome changes in stem/progenitor cell-derived EVs that may compromise their reparative function. In this study, we performed an unbiased analysis of EV transcriptome signatures from 3 different stem/progenitor cell types by RNA sequencing. The analysis revealed differential expression of a variety of RNA species in EVs. Specifically, we identified 241 common-dysregulated mRNAs, 21 ncRNAs, and 16 miRNAs in three stem cell-derived EVs. Gene Ontology revealed that potential function of common mRNAs mostly involved in metabolism and transcriptional regulation. This study provides potential candidates for preventing the adverse effects of hyperglycemia-induced stem/progenitor cell-derived EV dysfunction, and reference data for future biological studies and application of stem/progenitor cell-derived EVs.

## 1. Introduction

Stem/progenitor cell-based therapy has shown great promise in the treatment of a variety of diseases including myocardial infarction (MI), both in preclinical and clinical trials [1,2]. We and others have shown that bone marrow (BM)-derived endothelial progenitor cells (EPCs), cortical bone stem cells (CBSCs), and cardiac progenitor cells (CPCs) promote neovascularization and reduce fibrosis post-MI to attenuate ischemic injury [3,4,5]. However, autologous stem cell transplantation exhibits challenges such as low viability, low cell retention, and poor engraftment that lead to limited cardiac repair and regeneration [6,7]. Therefore, enhancing the efficacy of stem cell-based therapy is of utmost importance.

Recent evidence suggests that stem/progenitor cells secrete paracrine factors which benefit cardiac repair and regeneration post-cardiac injury [8,9]. EVs including exosomes, 30–100 nm extracellular nano-vesicles, are major regulators for local and remote cell–cell communication and cell signaling induction by transferring material including DNAs, RNAs, proteins, and lipids to recipient cells [10]. Recent reports show that EV/exosome therapy is an alternative stem cell-free strategy involved in tissue protection and regeneration post-injury [11,12,13]. However, EV/exosome quality and production are not static; their activity, components, and function are affected by stem cell origin [14]. Basal and genetic/stimulus-induced stress conditions such as inflammation and diabetes can trigger stem/progenitor cells to release inequivalent EVs, in terms of alteration of vesicle cargo and therefore, function [15,16].

Hyperglycemia is one of the important complications of diabetes associated with increased cardiovascular complications [17]. Clinical reports demonstrate that patients with diabetes have higher rates of cardiovascular rehospitalization, mortality, and major adverse cardiac events compared to non-diabetic patients [18]. In addition, others and we have shown that diabetes induces impairment of stem/progenitor cell function [19,20]. This may reflect the cellular dysfunction that is known to ensue in stem cells obtained from diabetic patients. For example, compelling evidence indicates that EPCs treated with high glucose (HG) or isolated from high-fat-diet mice represents a mechanism for impaired vascular repair and angiogenesis, subsequently leading to vascular dysfunction [21,22,23]. Limited studies focus on the effect of hyperglycemia on stem/progenitor cell- derived EVs. Therefore, understanding the molecular basis of stem/progenitor cell-derived EVs under hyperglycemic stress may be a strategy to enhance cell-free therapeutics for myocardial/ischemic tissue repair in diabetic patients.

To address this question, we determined the basis of hyperglycemic stimuli-induced EPC-, CBSC-, and CPC-derived EV transcriptome signatures. We performed comprehensive unbiased exo-next generation sequencing and bio-informatic analyses of EVs and found common transcripts (mRNAs, miRNAs, and ncRNAs) that were changed in EVs of three stem cell types from different origins in HG compared to normal glucose (NG) groups. Gene Ontology (GO) analysis further elucidated their function. Our data provide a wealth of information and we propose that modulation of potential target genes within EVs may rescue dysfunctional stem/progenitor cell-derived EVs for autologous therapies in diabetic patients with cardiac and other diseases.

## 2. Materials and Methods

### 2.1. Animals, Cell Isolation, and Culture

All animal procedures were performed following the approved protocols of the Institutional Animal Care and Use Committee, Temple University. Endothelial progenitor cells were isolated from 8–10-week-old C57BL/6J mouse BM from tibiae, femurs, and hip bones and cultured in endothelial cell basal medium-2 (EBM-2, Lonza) supplemented with growth factors (EGM-2 SingleQuots, Lonza) and 10% exosome-free FBS as previously described [15]. Media were collected every day from day 4 to day 10. Cardiac progenitor cells were isolated from mouse heart based on c-kit expression described previously and cultured in DMEM/F12 and neurobasal medium (1:1) supplemented with 10% exosome-free FBS, L-glutamine (2 mM), insulin-transferrin-selenium (ITS) (1%), penicillin-streptomycin (P/S), B27 (1%), N2 (1%), and growth factors (bFGF, EGF, and LIF) [24]. Cortical bone stem cells were kindly provided by Dr. Houser and cultured in DMEM/F12 supplemented with 10% exosome-free FBS, P/S, ITS, and growth factors (bFGF, EGF, and LIF) as previously described [3]. After isolation, all cells were cultured in parallel in normal or hyperglycemic (25 mM glucose) media in 37 °C, 5% CO_2_ atmosphere. Media were continuously collected after 48 h HG treatment followed by EV isolation.

### 2.2. EV Isolation and Characterization

EVs were collected from exosome-free FBS media of EPCs, CPCs, and CBSCs and isolated by ultracentrifugation method as previously described [25]. In brief, cell debris was removed by 13,000 rpm (20,000× *g*) for 30 min centrifugation followed by 30% sucrose cushion step at 35,000 rpm (120,000× *g*) for 60 min (Ti70 rotor, Beckman Coulter, USA). The interface was further spun at 35,000 rpm (120,000× *g*) for 60 min to pellet EVs. The purified EVs were dissolved in saline, aliquoted and stored in −80 °C. After isolation, EV particle number and size were characterized by Nanosight (NS300, Malvern) using Nanoparticle Tracking Analysis (NTA) software.

### 2.3. Cell/EV Lysate Preparation and Western Blotting

All procedures were carried out as reported previously [4]. Briefly, cell and exosome protein samples were collected in lysate buffer following BCA protein quantification. Samples were run by electrophoresis on Mini-PROTEAN TGX gels (Bio-rad Laboratories). Proteins were then transferred to nitrocellulose membrane and incubated overnight at 4 °C with primary antibodies of EV markers: Flotillin-1 (Abcam, ab41927; 1:1000) and TSG101 (Santa Cruz, sc-7964; 1:500), and β-actin (Cell Signaling, #4970L; 1:1000) as negative control. IRDye secondary antibodies (1:10,000) were used for protein detection using Li-Cor Odyssey imaging system.

### 2.4. Nanoparticle Tracking Analysis (NTA)

NS-300 Nanosight instrument (Malvern Instruments Ltd., Malvern, UK) equipped with a sCMOS camera (Hamamatsu Photonics, Hamamatsu, Japan) and a 405 nm laser was used. Data acquisition and processing were performed using NTA software version 2.3 build 0025. Background extraction was applied, and automatic settings were employed to determine the minimum expected particle size, minimum track length, and blur settings. Data were obtained at camera-level 12 (shutter: 600, gain: 350). Three movies of 30 s at 25 frames per second were recorded and assigned a single measurement in triplicates.

### 2.5. RNA Sequencing and Generation of RNA Sequencing Datasets

A total of 50 μg EVs was sent to System Biosciences (SBI) for exo-next generation sequencing. EV total RNA was isolated by SBI according to the manufacturer’s instructions using Sera Mir exosome RNA Purification Column kit (Cat #RA808A-1, System Biosciences, Palo Alto, CA, USA). The NextSeq High Output single-end sequencing ran at SR75 using NextSeq 500/550 High Output v2 kit (Cat #FC-404-2005, Illumina, San Diego, CA, USA) was performed according to the manufacturer’s instructions.

### 2.6. RNAseq Data Analysis

The sequence reads were analyzed using the EV whole transcriptome analysis RNA-seq Analysis Kit on the cloud-based Maverix Analytic Platform. Mouse genome mm10 were used for mapping using Bowtie [26], and raw sequence counts were enumerated. The database source for each RNA class was UCSC genome assembly mm10. The differential expression between samples across different conditions was statistically accessed by R/ Bioconductor package DESeq2 [27]. Genes with false discovery rate (FDR) < 0.05 and fold-change (FC) ≥ 2 were considered as significant. Pathway and network analysis were generated using Ingenuity Pathway Analysis (IPA) software. MicroRNA Target Filter within IPA was also used to perform microRNA target prediction which used the predicted microRNA-mRNA relationships from TargetScan and experimentally observed relationships from TarBase. GO enrichment analysis of the significant genes was performed using GOstats package in Bioconductor and *p* value < 0.01 cutoff was to claim significance [28]. Multi-dimensional scaling (MDS) plots and heatmaps were generated using custom scripts with R.

### 2.7. Statistical Analysis

All data were analyzed as mean ± S.E.M using GraphPadPrism7 (GraphPad Software, La Jolla, CA; USA); statistics were shown according to multiple t-test followed by the Bonferroni–Dunn method.

## 3. Results

### 3.1. Characterization of Stem/Progenitor Cell-Derived EVs

**Figure 1:**
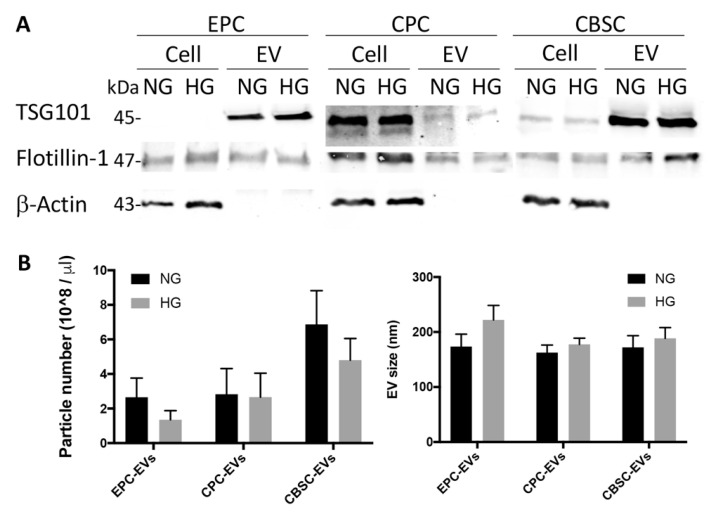
Endothelial progenitor cell (EPC)-, cardiac progenitor cell (CPC)-, and cortical bone stem cell (CBSC)-derived EV characterization in NG and HG conditions. (**A**) EVs were identified using EV protein markers, TSG101 and flotillin-1, and a negative marker, β-actin, by Western blot analysis. (**B**) EV particle size and exact particle number of NH and HG groups were measured by Nanosight. All Data shown as mean ± SEM.

EPCs, CBSCs, and CPCs isolated from mouse BM, cortical bone, and heart, respectively, were stimulated with 25 mM HG, mimicking the hyperglycemic condition, which is characteristic of diabetic patients. Isolation and characterization of EPCs, CBSCs, and CPCs were well-established in our lab as previous described and/or provided by our collaborator [3,15,24]. EVs collected from the equal amounts (10 µg) of NG and HG culture supernatants were examined by Western blot with different EV/exosome markers, TSG101 and Flotillin-1. As expected, the EV/exosome markers were presented in NG and HG EVs but not β-actin (a negative control) and there was no significant difference between groups (Figure 1A). To determine EV size and concentration, we performed NTA, NTA analysis revealed HG had no effect on mean particle size or concentration across different stem/progenitor cell-derived EVs (Figure 1B).

### 3.2. Hyperglycemia Significantly Alters Transcriptome in EPC-, CBSC-, and CPC-Derived EVs

**Figure 2:**
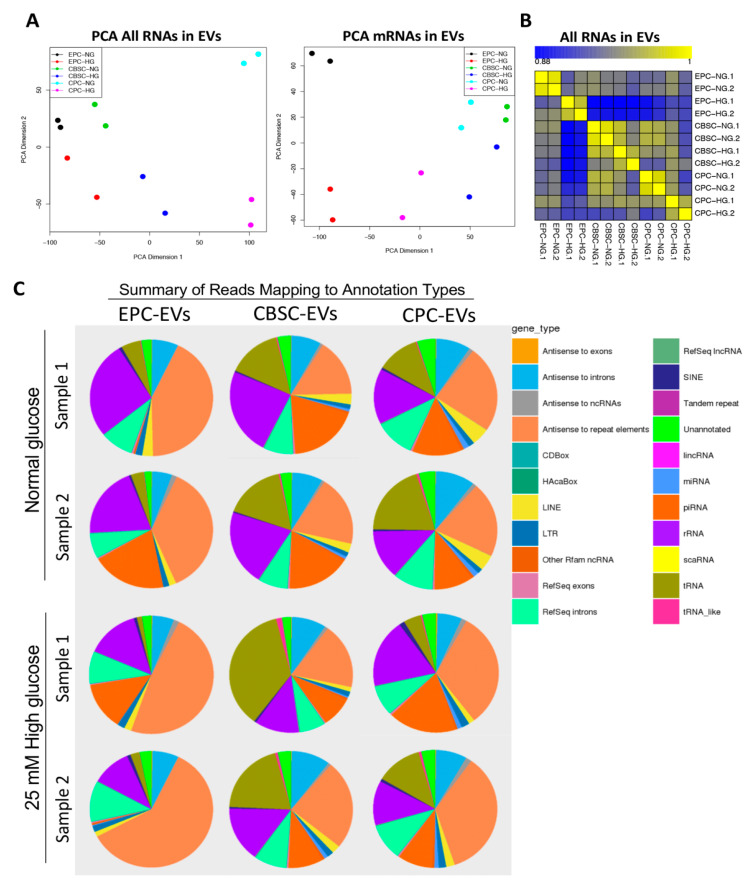
Whole transcriptome analysis of EPC-, CPC-, and CBSC-derived EVs and gene annotation types. (**A**) All EV RNA-seq data were processed using multi-dimensional scaling. Each dot represents a biological duplicate. (**B**) Pearson correlation of RNA-seq data. (**C**) Pie chart represents the fraction of each RNA type constitutes of total RNA content in EVs of EPC, CPC, and CBSC under NG and HG conditions.

Others and we have shown that stress alters stem/progenitor cell-derived EV content, which directly links to EV function/dysfunction [14,15,16]. However, if HG alters the content of different stem cell-derived EVs and if HG alters specific cargo irrespective of stem cell origin with common molecular determinants, triggering EV dysfunction is unknown. Our unbiased Exo-NGS analysis revealed differences in all the detected genes using MDS among 12 groups (EPC, CPC, and CBSC with two biological replicates for each under NG and HG conditions). Importantly, MDS plot clearly separated genes between NG and HG in all three stem/progenitor cell types. For biological duplicates, genes were clustered together, suggesting that stem/progenitor cell-derived EVs strongly reflected cell type-specific and hyperglycemia-induced gene expression patterns (Figure 2A). Pearson correlation further confirmed the high correlation of gene expression based on EV origin, and between NG and HG groups, indicating biological duplicates were in good accordance (Figure 2B). EV RNA-seq analysis revealed a large number of different RNA species within EVs. Portion of different types of RNA species was changed under HG stimulation, indicating linkage not only to stem cell types, but also responses to hyperglycemia (Figure 2C).

### 3.3. Gene Analysis of EPC-, CBSC-, and CPC-Derived EVs Under Hyperglycemic Conditions

**Figure 3:**
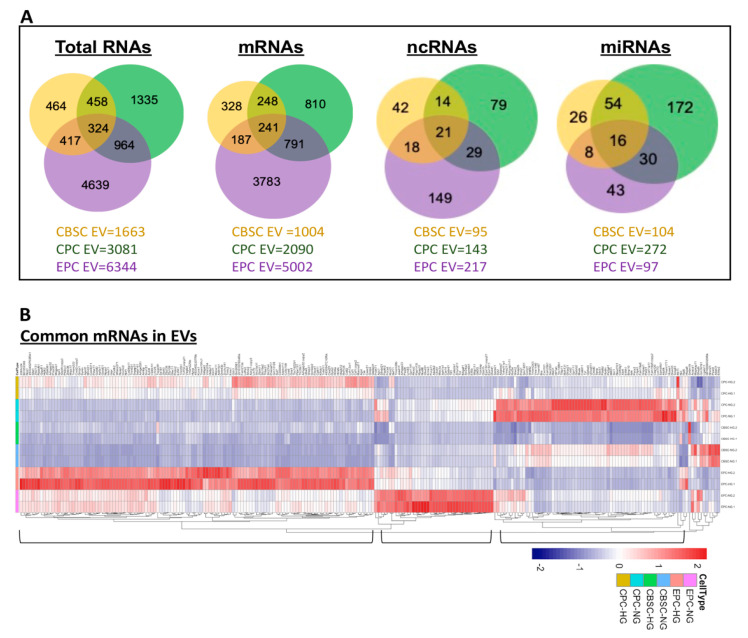
RNA-seq analysis of differentially expressed genes among hyperglycemic EVs. (**A**) Venn diagram representation of differential genes (only FC ≥ 2 and FDR < 5% were considered) of total RNAs, mRNAs, and non-coding RNAs: CBSC- (yellow), CPC- (green), and EPC- (purple) expressed genes, and common genes (grey) are shown. A total 324 genes (241 genes of mRNAs, 21 of ncRNAs, and 16 miRNAs) were presented in all stem cell types. (**B**) Heat maps show differentially expressed mRNAs. EV RNA-seq data were analyzed using DESeq2 package. Red indicates higher expression and blue indicates lower expression (n = 2 mice/group).

Multiple stem/progenitor cell-derived EVs are currently under testing in clinical trials (www.clinicaltrials.gov). In this perspective, unraveling a common set of abnormal genes in EPC-, CBSC-, and CPC-derived EVs under hyperglycemia may provide insights of potential target genes to enhance their therapeutic efficacy. Moreover, these results can be extrapolated to other stem cell EVs from diabetics to enhance their therapeutic efficacy. We performed differential analysis of each cell type separately using DESeq2 (NG vs. HG within each line). The detailed statistical results are provided in Supplementary Table S1. Our unbiased analysis revealed 324 differentially expressed genes (DEGs) in common among all three stem/progenitor EV preparations (Supplementary Table S2). Since RNAs occur in different forms and exhibit different regulatory roles, we segregated those common 324 RNAs into mRNAs (241 genes), non-coding RNAs (ncRNAs) (21 genes), and micro-RNAs (miRNAs) (16 genes) shown in Venn diagrams (Figure 3A). The remaining 46 RNAs were tRNA, piRNA, and other RNAs. Those common genes would be potential candidates for EV functional studies to rescue diabetic stem/progenitor cell-derived EV reparative properties. Heatmaps of mRNAs were generated using R package heatmap and hierarchical clustering analysis, based on Euclidean distance, to cluster samples. Differentially expressed mRNAs were categorized into 3 main clusters using FDR < 5% and FC ≥ 2 (Figure 3B).

### 3.4. GO Analysis and Networking of Common mRNAs in EPC-, CBSC-, and CPC-Derived EVs

**Figure 4:**
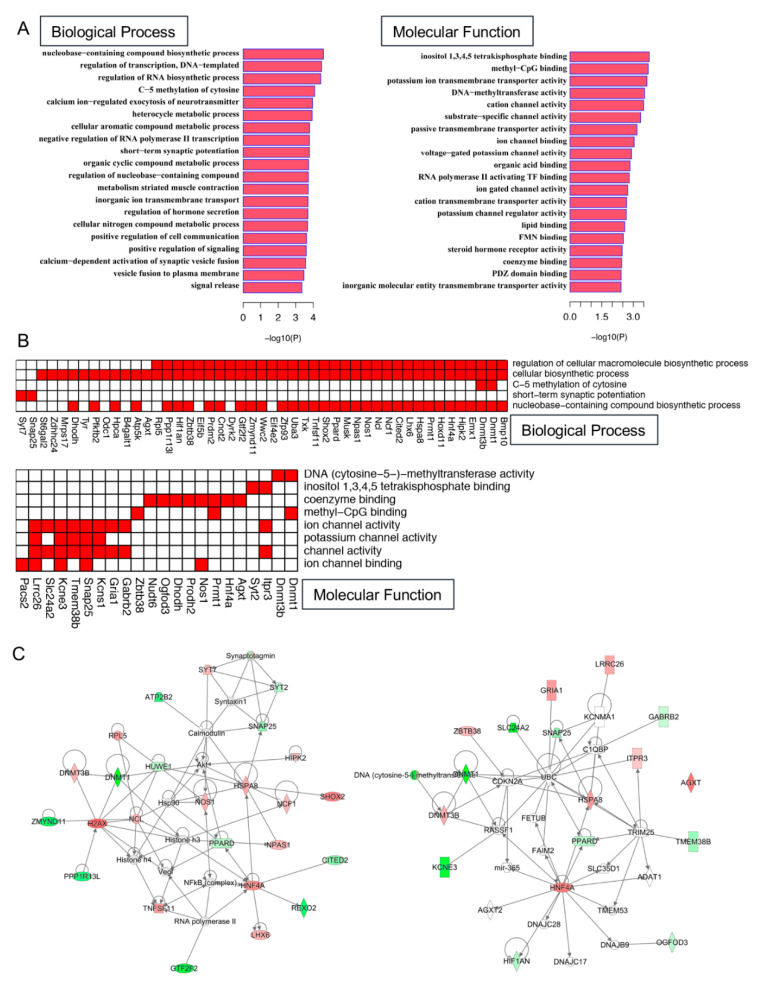
The GO prediction for biological process and molecular function of targeted EV mRNAs. (**A**) Top 10 significantly enriched categories in biological process (BP) and molecular function (MF) of genes common to all stem/progenitor cell-derived EVs. (**B**) Plot shows gene members within significantly enriched gene ontology categories in BP and MF of the dysregulated genes. (**C**) Gene interaction networks of significant genes. Left: genes from the top enriched gene ontology categories within biological process; Right: genes from the top enriched gene ontology categories within molecular function.

To further predict the function of differentially expressed genes (DEG)s in response to hyperglycemia, we performed the GO enrichment analysis and pathway/network analysis using IPA. Top enriched GO categories with the most significant *p* values (< 0.005) include both biological process (BP) and molecular function (MF). For BP, DEGs were enriched in biosynthetic processes, methylation of cytosine, regulation of transcription, and metabolic processes that were consistent with diabetic metabolic disorder [29,30]. For MF, DEGs were enriched in DNA methylation and channel activity (Figure 4A). Genes which fall into the most significant GO categories within BP and MF were indicated in Figure 4B. IPA analysis was performed to further provide insights into potential signaling pathways and genes (Supplementary Table S3). We found “Gene Expression”, “Cell Death and Survival”, “Cellular Function and Maintenance”, “DNA Replication, Recombination, and Repair”, and “Nucleic Acid Metabolism” were enriched. To have a better understanding of the interaction of genes, we also performed the gene interaction network analysis using genes from these top enriched gene ontology categories (Figure 4C—left for genes in top BP, and right for genes in top MF categories).

### 3.5. Common miRNAs and Basic Information of LncRNAs in EPC-, CBSC-, and CPC-Derived EVs

**Figure 5:**
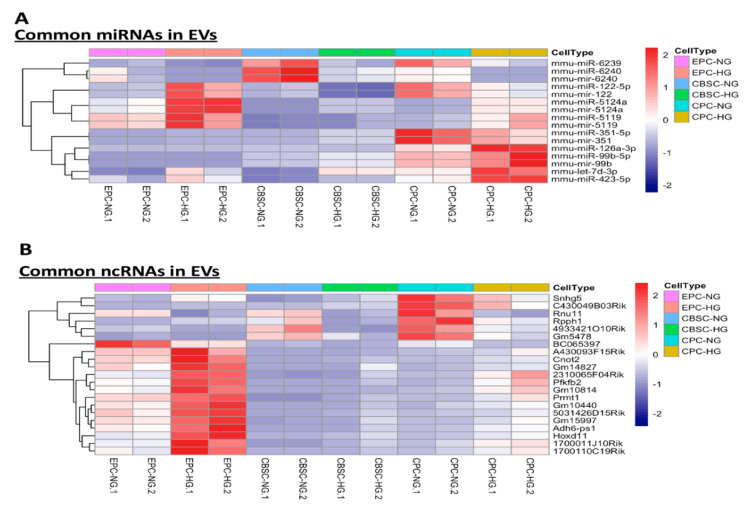
Heatmap of differentially expressed common miRNAs and ncRNAs among hyperglycemic EVs. (**A**) Heat map shows differentially expressed miRNAs. (**B**) Heat map shows differentially expressed ncRNAs.

It has been reported that miRNAs are selectively sorted into stem/progenitor cell-derived EVs and play important roles in mediating physiological and pathological processes of cardiovascular diseases [15,31,32]. Investigation of dysregulated miRNAs in stem/ progenitor cell-derived EVs under hyperglycemic insult may provide potential therapeutic targets for rescuing EV reparative function. We identified 16 miRNAs, and only 8 of them changed in the same direction (upregulated miRNAs: miR-5124a, miR-5119, miR-99b-5p, let-7d-3p, and miR-423-5p; downregulated miRNAs: miR-6239, miR-6240, and miR-351-5p) among all three cell sources of EVs from stem/progenitor cells subjected to HG (Figure 5A). Subsequently, TargetScan was used to assess the differentially expressed miRNA-targeted mRNA. There are only 7 miRNAs that can be predicted to target a specific mRNA. Mmu-miR-423-5p was predicted to target more than 1881 mRNA. (Supplementary Table S4). These reference data provide miRNAs that may be targeted for reversing hyperglycemia-induced stem/progenitor cell-derived EV dysfunction and for the discovery of the underlying signal transduction pathways by the miRNA-target mRNA list. The predicted targets also identified as differentially expressed genes from the exosome of each cell type (Supplementary Table S5). Moreover, recent studies have focused on ncRNAs, especially long non coding RNAs (lncRNAs), which play important roles in epigenetic, transcriptional, and translational regulation [33,34]. However, ncRNAs in stem/progenitor cell-derived EV have not been well studied for their expression changes or biological functions in response to hyperglycemia. Our screening revealed 21 novel lncRNAs (Figure 5B) and their basic information is provided in Table 1 according to Noncode, NCBI, Ensembl, and RNAcentral databases.

## 4. Discussion

Stem/progenitor cell-based therapy has emerged as a promising strategy for cardiac repair and regeneration [1,2]. Various stem/progenitor cell types including BM-derived stem/progenitor cells and cardiac-derived stem/progenitor cells are currently used in clinical trials for cardiovascular diseases. However, the clinical outcomes are at best, modest. Recent studies and those from our labs suggest that EVs largely recapitulate the reparative activity of the stem/progenitor cell themselves in ischemic tissue repair and regeneration. Specifically, EPC-, CPC-, and CBSC-secreted EVs benefit cardiac repair and regeneration following ischemic injury [4,35]. However, EV cargo and biological properties differ depending on physiological condition of their parental cells [12,13,14]. Different biological content such as DNAs, RNAs, and proteins are selectively sorted into EVs, while stem/progenitor cells are under physiological conditions or with stress. It remains unknown if the CVD prone factor, hyperglycemia, which occurs in diabetic patients, alters EV components and compromise their function. A better understanding of the change of molecular component in EVs in response to hyperglycemia may help with the identity of molecular targets that may be altered to rescue diabetic stem cell-derived EV functions and improve the efficiency of stem/progenitor cell-derived EV approach on ischemic injury in diabetic patients.

In this study, we performed an unbiased Exo-NGS RNA sequencing, to unravel EV mRNAs, miRNAs, and ncRNAs, which are commonly dysregulated in response to hyperglycemia in three different stem/progenitor cell types. According to mRNA GO and IPA analysis, we found that gene expression and transcriptional regulation are those of most enriched function. Several epigenetic modifying genes (*Dnmt1*, *Dnmt3b*, and *Prmt1*) were identified in both enrichments. *Dnmt*, encoding for DNA methyltransferases, is important for CpG island methylation and may cause transcription silencing [36]. In agreement with above reports, our RNA-seq data showed that *Dnmt1* was decreased and *Dnmt3b* was increased consistently in all three stem/progenitor cell-derived EVs subjected to hyperglycemia. In line with our findings, Vigorelli et al. reported that HG also induced *Dnmt1* downregulation and *Dnmt3b* overexpression in both mRNA and protein levels in human CD34^+^ stem cells, and CpG island and non-CpG island are hypermethylated at CXCR4, promoter [37]. As *Dnmt1* and *Dnmt3b* exhibit similar expression pattern under HG treatment in both mouse and human models, it provides more translational relevance to alter their expression for enhancing stem/progenitor cell- derived EV function.

In addition to mRNAs, ncRNAs including miRNAs and lncRNAs without protein coding potential have gained a lot of interest due to their regulatory roles in reparative processes of injured heart [32,38,39,40]. Moreover, RNA-sequencing data reveals that ncRNAs are selectively sorted into exosomes under stimulation [41,42]. Intensive studies have demonstrated that miRNAs enriched in stem/progenitor cell-derived EVs facilitate cardiac repair post-MI [32,43,44]. However, none of them focused on the effect of hyperglycemia insult, exclusively. In this study, we identified HG-induced 16 common miRNA deregulation in three different types of stem/progenitor cell-derived EVs. Among them, previous studies also showed that miR-122-5p expression level is changed in response to diabetes. For example, serum miR-122-5p was significantly increased in patients with T2DM and patients with diabetic retinopathy, and was sorted into serum EVs in the gestational diabetes mellitus case [45,46]. Even though we only found miR-122-5p was increased in BM-EPC-derived EVs, the most used cell type in clinical trials, but not other two cell types that may reflect specificity of response in a cell-specific manner. Moreover, another miRNA, miR-423-5p, was indicated as a biomarker for diagnosis of heart failure and was involved in inhibition of cancer proliferation [47,48]. In our study, we found miR-423-5p was increased in all three HG-induced EVs. This evidence suggests that miR-423-5p might play a pivotal role in HG- induced stem/progenitor cell dysfunction.

Along with miRNAs, lncRNAs have emerged as important gene regulators in terms of RNA decoy: repelling ribonucleic protein away from chromatin; recruitment: recruiting chromatin modifiers to specific DNA motifs and RNA sponge: stabilizing mRNAs by binding miRNAs [49,50]. Limited studies have focused on lncRNAs in stem/progenitor cell-derived EVs in cardiac repair especially when subjected to hyperglycemia [51]. To the best of our knowledge, we are the first to screen common stem/progenitor cell-derived EV lncRNAs that are responsive to hyperglycemia. Of note, despite the importance of lncRNA involvement in multiple cellular processes, most lncRNAs we screened have not been annotated and their functions are unknown. We narrowed down the potential lncRNA candidates that can be further studied biologically and may be novel therapeutic targets. In corroboration to our findings, a recent study reported that lncRNA-snhg5 expression was reduced in blood samples from type II diabetic patients after 6 months of sleeve gastric surgery. lncRNA-snhg5 expression was induced in epithelial cells under HG/high fat conditions, and inhibition of lncRNA-snhg5 reduced epithelial cell dysfunction in vitro [52]. Mechanistically, lncRNA-snhg5 acts as a miRNA sponge and plays a role in promoting cancer proliferation and migration. Therefore, we speculate that reduction of this lncRNA can help reverse reparative function of HG-induced EVs. Mao et al. reported that another candidate, lncRNA-Cont2 and its neighbor gene, Cont2, were involved in inflammation in BM-derived macrophages [53]. As known, diabetes exhibits chronic inflammation and in corroboration with the above reports, we found lncRNA-Cont2 was upregulated in EVs of all three stem/progenitor cell types under hyperglycemic condition. This may indicate that HG-induced-stem/progenitor cells EV lncRNA-Cont2 triggers inflammation under hyperglycemic condition and may promote cardiac dysfunction.

Like any in silico, unbiased study of this nature, there certainly are some limitations in this study. Identified genes of interest needs to be validated and their functional role needs to be established in cell biology and in vivo in the ischemic heart injury models of diabetic animals. Additionally, only stem cell-derived exosomes from mice were analyzed; therefore, it would be necessary to confirm exosomal RNA expressions in the human system to obtain more translational meanings. We also did not analyze other RNAs such as piRNAs and snoRNAs, which may play critical role in gene regulation.

## 5. Conclusions

Taken together, this study provided an unbiased transcriptome profile, especially common mRNA, miRNA, and lncRNA molecular signatures in EPC-, CPC-, and CBSC-derived EVs under hyperglycemic condition. This data may benefit the identification of transcripts that may be targeted to reverse diabetic stem/progenitor cell-derived EV dysfunction on one hand and provide guidance to future biological studies looking at mechanisms of diabetic EV function/dysfunction on the other.

## Figures and Tables

**Table 1:** Common differentially expressed ncRNAs identified in EPC-, CPC-, and CBSC-derived EVs in hyperglycemia.

Gene Name	Chromosome	Length	Strand	Exon	Start	End
Snhg5	Chr9	1010	−	5	88521052	88522897
C430049B03Rik	ChrX	2993	−	4	53053111	53057190
Rnu11	Chr4	134	−	1	132270079	132270186
Rpph1	Chr14	325	−	1	50807446	50807771
4933421O10Rik	Chr4	3850	−	2	33027138	33031323
Gm5478	Chr15	2960	−	-	101643020	101647380
BC065397	ChrX	3243	+	10	136741824	136803364
A430093F15Rik	Chr19	2115	+	4	10740946	10786043
Cnot2	Chr7	553	−	4	59556901	59587385
Gm14827	ChrX	3089	+	4	94442731	94447727
2310065F04Rik	Chr11	2724	−	5	67112460	67120080
Pfkfb2	Chr13	383	−	3	47421808	47423198
Gm10814	Chr19	1966	+	5	6012619	6018459
Prmt1 (H)	Chr4	1402	−	1	147615843	147617245
Gm10440	Chr5	2753	+	4	54349990	54358543
5031426D15Rik	Chr2	6021	−	1	6922701	6928722
Gm15997	Chr5	1491	−	5	149489055	149538030
Adh6−ps1	Chr3	-	+	4	138374121	138388291
Hoxd11	Chr2	-	+	-	74679558	74687016
1700011J10Rik	Chr2	1147	+	-	72979432	72989249
1700110C19Rik	Chr17	722	+	3	10324601	10329312

## Supplementary Materials

The following are available online at https://www.mdpi.com/2073-4409/9/9/2098/s1, Supplementary Table S1: Total reads and read number of each RNA category. Supplementary Table S2: Differential expressed 324 genes. Supplementary Table S3: IPA analysis for potential signaling pathways and genes. Supplementary Table S4: Prediction of miRNA-targeted mRNAs. Supplementary Table S5: Predicted differentially expressed targets in exosomes.
